# Effects of Emulsifier Type and Post-Treatment on Stability, Curcumin Protection, and Sterilization Ability of Nanoemulsions

**DOI:** 10.3390/foods10010149

**Published:** 2021-01-13

**Authors:** Rui Li, Qiangsheng Fang, Peihong Li, Chunling Zhang, Yuan Yuan, Hong Zhuang

**Affiliations:** 1 School of Materials Science and Engineering, Jilin University, Changchun 130022, China; ruili18@mails.jlu.edu.cn (R.L.); fangqs19@mails.jlu.edu.cn (Q.F.); liph18@mails.jlu.edu.cn (P.L.); 2 College of Food Science and Engineering, Jilin University, Changchun 130062, China; yuan_yuan@jlu.edu.cn (Y.Y.); zhuanghong@jlu.edu.cn (H.Z.)

**Keywords:** nanoemulsions, curcumin, stability, emulsifier type, photodynamic inactivation (PDI)

## Abstract

Curcumin has a high inhibitory effect on many potential diseases caused by bacteria and fungi. However, its degradability and low water solubility limit its application. Loading curcumin with an emulsion delivery system can overcome these problems. Five different types of emulsifiers were used to prepare the curcumin-loaded nanoemulsions, namely, Tween 80 (T80), Span 80 (S80), sodium dodecyl sulfate (SDS), soybean protein isolate (SPI), and lecithin (LEC). The effects of emulsifier types and post-treatment methods on emulsion stability and curcumin-load efficiency were studied. In addition, photodynamic inactivation was used to test the antibacterial effect of nanoemulsions on *Escherichia coli* under blue light excitation. The five types of emulsifiers could form uniform emulsions with good storage stability and with antibacterial capacity on *Escherichia coli*. Among them, the T80 and LEC emulsions had good stability, coating effect, and sterilization performance under heating or room temperature. Both curcumin-loaded bactericidal emulsions had the potential for large-scale applications. A nanoemulsions delivery system could effectively improve the dispersion and chemical stability of curcumin in water. An emulsion loaded with antibacterial photosensitizer represents a new idea for the storage and preservation of food commodities.

## 1. Introduction

Curcumin is a natural polyphenol extracted from the rhizomes of ginger plants. Commercially available curcumin is usually composed of a mixture of curcumin and its structurally related compounds: demethoxycurcumin and bisdemethoxycurcumin, which are collectively referred to as curcuminoids [1]. Among the three types of curcuminoids, curcumin has the most potent antioxidant, bactericidal, anti-inflammatory, and anti-cancer effects, as well as other drug properties, because of the presence of the methoxy group on the benzene ring [2]. At the molecular level, the interaction between curcumin and other chemicals is based on the unique chemical properties of curcumin, including the ability to give and receive hydrogen bonds, the ability to bind to cations, the rotation of CC bonds, and the ability of Michael acceptors [1]. In addition, curcumin has good biocompatibility and food safety as a natural plant extract. Clinical trials have shown that its single oral dosage can reach 12 g/day [3], and it has extremely low toxicity. Curcumin has in vitro antibacterial potential against various microorganisms, such as fungi and several Gram-positive and Gram-negative bacteria [4,5,6]. Curcumin can kill Gram-positive (*Staphylococcus aureus* and *Enterococcus faecalis*) and Gram-negative bacteria (*Escherichia coli* and *Pseudomonas aeruginosa*), and its antibacterial capacity increases with increasing dose and incubation time. A dose of 100 μM curcumin still has a 100% killing effect on bacteria with a density of 10^6^ CFU/mL [6]. Curcumin can be used as a bactericidal agent or photosensitizer to utilize the photodynamic inactivation (PDI) reaction mechanism to achieve a bactericidal effect. Studies have shown that curcumin-mediated PDI can effectively inactivate *Listeria monocytogenes* cells and eliminate their mature biofilms [7]. Therefore, the curcumin-mediated PDI treatment can effectively kill some food-borne bacteria, thereby greatly improving the antibacterial effect on food in the process of sterilization and preservation.

Although curcumin has a high inhibitory effect on many potential diseases caused by bacteria and fungi, curcumin’s low water solubility and easy degradation limit its application and development. Curcumin, as a strongly hydrophobic molecule, has a water solubility of only 3 × 10^−8^ M [8], and it will rapidly oxidize under neutral or alkaline conditions. The oxidation products (bicyclopentadione and others) [9] reduce the utilization rate and drug efficacy. An obvious way to improve curcumin drug availability is to use nano-delivery systems, such as polymer nanoparticles, micelles, liposomes, hydrogels, and emulsions [3,10], to increase its water solubility. Such systems can be used in drug delivery/targeting directions. Some nanocarrier systems have reached clinical evaluation and application. Bucurescu et al. [11] used gum arabic as an encapsulating agent and spray-drying method to microencapsulate curcumin. Wu et al. [12] prepared the β-cyclodextrin /CUR complex by wrapping curcumin with β-cyclodextrin, which significantly improved the water solubility of curcumin.

Recent research shows that the encapsulation method of bioactive compounds by nanoemulsions is applicable to the food industry. Nanoemulsions are the main nanocarrier systems used to encapsulate and deliver curcumin [1]. The droplet size of the nanoemulsions is very small with an average radius of about 50 to 200 nm; thus, it has higher stability than conventional emulsions [13]. The preparation methods of nanoemulsions can be divided into the following: high-intensity methods, such as high-speed mixing, high-pressure homogenization, high-pressure microjet, and ultrasonic vibration; and low-intensity methods, such as spontaneous phase inversion and microemulsion dilution [14,15]. According to the different emulsifier and curcumin solvent selected, food grade nanoemulsions with high stability, bioacceptability, and bioavailability can be prepared. The preparation of curcumin-loaded nanoemulsions can effectively improve the shortcomings of curcumin’s low water solubility and stability, thereby maximizing curcumin’s characteristics as a photosensitizer and allowing it to be used in food sterilization and preservation.

Many factors affect the properties of emulsions, such as the selection and ratio of the oil and water phases, emulsifier type, dosage, homogenization conditions, ionic strength, pH conditions, and others [16]. The stability of the emulsion depends on the composition and formulation. Thus, the type of emulsifier is a key factor in stabilizing the system. The emulsifier kinetically stabilizes the emulsion by reducing the interfacial tension between the oil and water phases and by forming a film at the oil in water interface, thereby suppressing the demulsification of the dispersed lipid droplets. The ability of emulsifiers to reduce interfacial tension usually comes from electrostatic repulsion and space repulsion among emulsifiers [13,17]. Different emulsifiers have different stability levels to emulsions. The emulsifying ability of small molecule surfactants is usually determined by different factors, such as the size of their polar head groups and the chain length of non-polar tail groups. The functional properties of phospholipids depend on the nature of the head and tail groups and the unsaturation of the two fatty acid chains. The spherical or flexible structure of the protein affects its adsorption properties at the droplet interface [16].

The emulsion used in the food industry undergoes various processes, such as mixing, shearing, heating, freezing, among others. The storage of the prepared emulsion can be affected by the storage temperature and light. These complex physical processes cause instability of the emulsion and the decomposition of biologically active substances in varying degrees. In this sense, Zarena et al. [18] found that the emulsion treated by heat (70 °C) and NaCl (100 and 200 mM) showed creaminess and droplet aggregation after 60 days of storage.

The thermal stability of various emulsifiers significantly differs according to their structures and molecular weight characteristics. Protein denaturation, unfolding [19], violent migration of small molecules, and intermolecular interactions may occur during heating. As a storage method that restricts the growth of microorganisms, maintains chemical stability, and prolongs the shelf life of foods [20], freezing generally adversely affects the stability of emulsions through various physical and chemical mechanisms, including lipid crystallization, ice formation, conformational changes of biological polymers, interfacial phase change, migration of small molecule emulsifier, damage of interfacial film, and others [21,22]. Therefore, choosing the right emulsifier is important for food manufacturers to reduce or avoid the problem of emulsion instability.

In this paper, the high-speed shear-high-pressure homogenization technique was used to prepare curcumin-loaded nanoemulsions. Compared to long-chain and short-chain triacylglycerols, medium-chain triglycerides have the highest bioavailability of curcumin, so it is selected as the oil phase [23]. Food emulsifiers, namely, Tween 80 (T80), Span 80 (S80), soybean protein isolate (SPI), and lecithin (LEC), and an indirect food additive sodium dodecyl sulfate (SDS) were used as emulsifiers. The effects of emulsifier types and post-treatment methods (room temperature, light, heat treatment, and freeze-thaw treatment) on emulsion stability and curcumin coating effect were studied. PDI technology was used to test the antibacterial effect of the nanoemulsions on *E. coli* under the excitation of a 460 nm blue light-emitting diode (LED) lamp, thereby explaining the potential application of the photosensitizer nanoemulsions in food packaging and food preservation. This article discussed the effects of food-grade emulsifier types and storage methods on the stability and loading efficiency of nanoemulsions, which could provide a technical reference for the preparation of nanoemulsions and contribute to their application and development.

## 2. Materials and Methods

### 2.1. Materials

Medium-chain triglyceride (MCT) oil was obtained from Shanghai YouChuang Industry Co., Ltd., Shanghai, China. MCT consists of caprylic acid triglyceride and capric acid triglyceride (59.1% and 40.7%, respectively). Span 80 (S80), lecithin (LEC), sodium dodecyl sulfate (SDS), Tween 80 (T80), and curcumin (CUR) (≥65%) were purchased from Aladdin Industrial Corp., Shanghai, China. Food-grade soybean protein isolate (SPI) and *E. coli* were prepared in the laboratory. The blue LEDs (455–460 nm, 60 cm, and 8 W) were purchased from Zhongshan China (Wanjia Lighting Co., Ltd., Shenzhen, China). All solvents and reagents were used directly without further purification.

### 2.2. Preparation of Curcumin Emulsions

Excess curcumin was added to a weighed amount of MCT. Then, the resulting mixture was stirred for 3 h (60 °C, 1200 rpm). The mixtures were centrifuged (9000× *g*, 10 min, room temperature) to remove the curcumin crystals. The supernatant was collected as an oil phase. The concentration of curcumin in MCT after dissolution and centrifugation is approximately 5.3 mg/mL.

A certain amount of emulsifier was weighed to dissolve in the oil phase (S80 and LEC) or deionized water (T80, SDS, and SPI) according to its solubility. A stock emulsion was prepared by homogenizing the oil and aqueous phases together with a high-speed blender (5000 rpm, 10 min). The resulting coarse emulsion was then passed through a high-pressure homogenizer (AH-NANO, High-pressure homogenizer, ATS, Brampton, Canada) at 1036 bar for 15 cycles. The final emulsion contains 2 wt.% emulsifiers and 15 wt.% oil phase. Sample emulsions were stored in the darkness at room temperature (25 °C).

### 2.3. Freeze-Thaw Treatment

Each cycle of freeze-thaw treatment consisted of freezing the samples at −20 °C for 24 h and thawed the samples at room temperature for 3 h. The process was repeated thrice. Besides, the digital photos of samples were taken before and after freeze-thaw treatment.

### 2.4. Heat Treatment

Equal amounts of different emulsion samples were heated in an oven at 60 °C, 90 °C, and 120 °C for 1, 2, 3, 4, 6, and 8 h, respectively.

### 2.5. Particle Size and Charge Measurements

The particle size distribution and Polydispersity Index of nanoemulsions were determined using DLS (Zetasizer Nano ZS-90, Malvern Instruments, Worcestershire, UK). The nanoemulsions samples were diluted 50 times in deionized water. Diluted emulsions were placed into disposable polystyrene cells (DTS0012, Malvern Instruments). Data were reported as the mean droplet diameter (hydrodynamic diameter). Polydispersity index is dimensionless and indicates the heterogeneity (monodisperse or polydisperse) of the sizes of particles in a mixture.

The droplet charge (zeta potential) of the nanoemulsions was determined using a particle micro-electrophoresis instrument (Zetasizer Nano ZS-90, Malvern Instruments, Worcestershire, UK). The samples were diluted 100 times in deionized water before measurement and the diluted emulsions were placed into disposable capillary cells (DTS 1060, Malvern Instruments).

Corresponding particle size measurement and potential measurement were performed on the samples after storage, illumination, freeze-thaw treatment, and heat treatment. All samples were measured at 25 °C in duplicate, with three readings for each measurement. The results were given as the average ± standard deviation of the six values obtained.

### 2.6. Curcumin Concentration Measurements

Dissolved 6 mg of CUR in 100 mL of absolute ethanol, diluted a part of the solution to a certain concentration (1, 2, 4, 8, 12, 16 µg/mL), and measured the absorbance at a wavelength of 425 nm using an ultraviolet spectrophotometer (UV-6100S, Mapada, Shanghai, China). For the standard curve of curcumin dissolved in ethanol (Figure S1).

Diluted the curcumin emulsion 100 times with absolute ethanol, measured the absorbance value, and calculated the curcumin content in the emulsion through the standard curve. Each sample was measured three times in parallel. Curcumin-load ratio was calculated using the following formula:

Curcumin-load ratio = C_1_ / C_0_ × 100%, (1)

where C_1_ is the concentration of curcumin in the emulsion; C_0_ is the initial concentration of added curcumin.

### 2.7. Confocal Laser Scanning Microscopy (CLSM)

Morphological analysis of the curcumin was performed using a CLSM. Moreover, 30 µL of the sample was loaded on a concave glass slide and covered with a glass cover slip (0.17 mm thickness). The sample was observed by CLSM (Zeiss LSM700 inverted, Germany) and were scanned at room temperature (25 ± 1 °C) using a 20×/0.5 objective lens. Auto-fluorescence from the crystals was excited using 488 wavelength lasers for curcumin crystals.

### 2.8. Experimental Procedure of PDI Inactivation

The distance between the LED light source and the *E. coli* solution in the 96-well plate was adjusted to 10.0 cm, and the brightness was 25,000 lux as measured by the illuminance meter. The irradiation times of the sample are 0, 5, 10, and 30 min, respectively.

The *E. coli* stored in the refrigerator at −80 °C was taken out, and a line was drawn after recovery. A single colony was mixed with 50 mL of lysogeny broth (LB) liquid culture medium, and the culture was placed in a constant temperature shaker at 37 °C for 8 h until the logarithmic period. The bacterial solution (100 μL) was mixed with different amounts of photosensitive emulsion and shaken. After dark incubation for a period of time, a LED blue light device was used to irradiate it for different times. The contents of the photosensitizer in the mixed solution were 0, 100, 150, and 200 μM. After the photodynamic treatment, the bacterial solution-emulsion mixture was diluted 10 times with phosphate buffer saline (PBS) to each group (10^−1^–10^−11^ times of the original treatment solution); 5 μL of each group was cultured on LB solid medium and incubated at 37 °C for 24 h. The total number of colonies was counted, and each dilution gradient was repeated. The obtained value was multiplied by the dilution factor, the counting unit was CFU/mL, and the antibacterial efficiency was calculated by the following formula:

Viability (%) = (CFUcontrol − CFUsample)/CFUcontrol × 100%,(2)

where CFUcontrol is the number of colonies in the blank without light and CFUsample is the number of colonies after photodynamic treatment.

### 2.9. Statistical Analysis

All experiments were carried out in three independent trials at least. The mean and the standard deviations were calculated. Statistical comparisons were made using Student’s *t*-test. The *p*-value < 0.05 was considered to be significant.

## 3. Results

### 3.1. Impact of Emulsifier Type on Storage Stability of Emulsions

**Figure 1:**
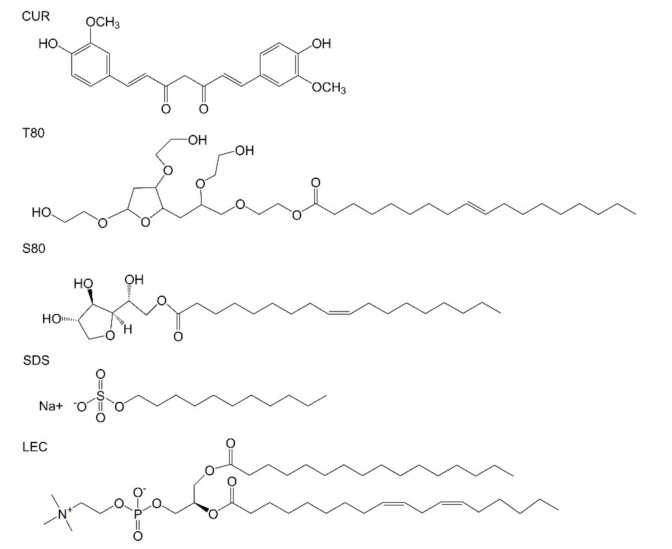
Molecular structure of curcumin and various emulsifiers (Tween 80 (T80), Span 80 (S80), sodium dodecyl sulfate (SDS), lecithin (LEC)).

Five kinds of curcumin-loaded emulsions were prepared with different emulsifiers, and the changes in droplet diameter and surface potential were observed within a week. The effect of different emulsifiers on the stability of the emulsion was explored. In addition, the coating effects of emulsion on photosensitizer were compared within 2 weeks under dark and light conditions. The molecular structure of each important compound is shown in Figure 1. The size distribution of the relevant emulsion is shown in Figure S2.

Table 1 shows that the average droplet diameter of emulsions stabilized by T80, SDS, SPI, and LEC was maintained at about 150 nm to 200 nm, whereas S80 had the largest average particle size of stable emulsions due to the poor hydrophilicity of the head. In addition, the dispersion index of the emulsion with S80 and SPI as emulsifiers was growth, indicating that the distribution of droplet diameter was more dispersed. The difference in the initial particle size of each emulsion is related to the size and hydrophilicity of the polar head group of the emulsifier and the steric hindrance provided by the emulsifier [13,24]. The SDS with the smallest head group and the strongest hydrophilicity provided the smallest droplet diameter. By increasing storage time, almost no significant change was observed, except for the emulsion with SPI as an emulsifier, thus indicating that these emulsions had good stability. The droplet diameter of the SPI emulsions increased the most, and demulsification occurred, indicating the worst stability. The initial droplet diameter of the SPI emulsion was about 200 nm. After 3 days, the droplet diameter showed a three-peak distribution, at 190, 838, and 5292 nm. After 7 days, the droplet diameter increased to about 4400 nm. The protein adhered to the inner wall, and the viscosity of the emulsion increased. The demulsification phenomenon of SPI emulsion might be due to the hydrophobic interaction between the amino acid residues of the polypeptide chain during storage [25], partly forming a gel-like structure [26], which can partially be flocculated in the form of large particles inside the water phase [27].

The surface potentials of emulsions stabilized by various emulsifiers significantly differed. SDS is an anionic surfactant, and the surface potential of the droplets of the stabilized emulsion was about −50 mV or less. The surface potentials of the rest of the emulsions were around −30 mV. Increasing the surface charge of droplets can slow down the emulsification rate because of the repulsive force between droplets increases [28]. If the surface potential value is above 30 mV, then the emulsion is stable [13]. Thus, these five types of emulsions had good stability under dark storage conditions at room temperature. Beattie and Djerdjev [29] suggested that the negative surface charge of the nanoemulsions droplets stabilized by the nonionic emulsifier is because the polyoxyethylene group carried by the nonionic emulsifier forms a hydrogen bond with the OH^−^ in the water, thereby inducing the adsorption of the OH^−^ on the oil–water interface. Roger and Cabane [30] hypothesized that the OH^−^ in water and the impure fatty acid in the oil phase have an acid-base neutralization reaction, and the formed fatty acid ions are adsorbed on the oil–water interface, which increases the number of charges carried by the droplet. However, with increasing storage time, the net value of the droplet surface potential of all emulsions decreased, indicating that the stability of the emulsions gradually decreased during storage. The surface potential value of the SPI emulsion decreased significantly with time, which was consistent with the results obtained for droplet size.

**Figure 2:**
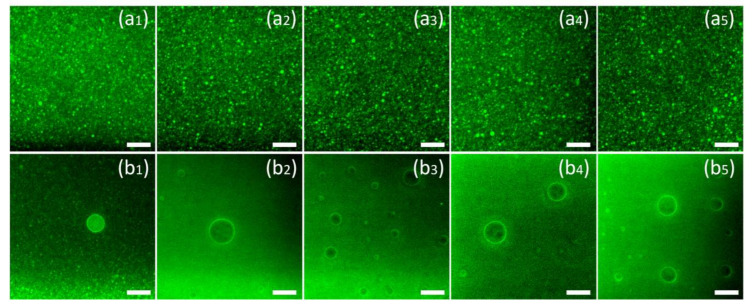
The Confocal Laser Scanning Microscopy (CLSM) image of the nanoemulsions using T80 (**a_1_**,**b_1_**), S80 (**a_2_**,**b_2_**), SDS (**a_3_**,**b_3_**), SPI (**a_4_**,**b_4_**), LEC (**a_5_**,**b_5_**) as emulsifiers. The brightness in the images is caused by the auto-fluorescence of curcumin (488 nm excitation). The scale bar is equal to 10 μm.

Figure 2 presents the laser microscope image of nanoemulsions prepared with different emulsifiers. The brightness in the image is caused by the autofluorescence of curcumin (excited at 488 nm). Figure 2a are the pictures of the nanoemulsions under observation. To clarify the presence of curcumin in the oil phase, micron-level droplets were selected for observation in the emulsion, as shown in Figure 2b. According to Figure 2a, it can be found that different emulsifiers will not cause differences in the distribution of curcumin. Curcumin mainly exists in the oil phase of the nanoemulsions and is evenly distributed inside the emulsion with the droplets. According to Figure 2b, it can be found that although curcumin is ubiquitous in the oil phase, significant aggregation occurs at the oil–water interface, which can be found by the increase in fluorescence intensity at the interface.

**Figure 3:**
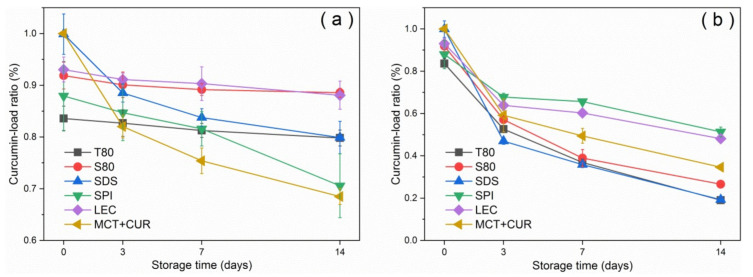
Variation of curcumin-load ratio with storage time under dark conditions (**a**) and light conditions (**b**) at 25 °C.

Figure 3 shows the curcumin-load ratio and error bars of the emulsion with storage time under dark and light conditions at room temperature. The curcumin-load ratio could reach more than 80% under dark conditions, whereas under light conditions, the photosensitizer degraded significantly. After 2 weeks of storage, T80 had the worst coating performance under dark conditions, as shown in Figure 3a. This result was due to the characteristics of neutral surfactants, which resulted in a higher affinity for curcumin compared with ionic surfactants. Curcumin was mostly located at the oil–water interface [31]. The curcumin molecule would be inside the T80 core-shell micelles [32] as it moved into the aqueous phase and underwent hydrolysis, which manifested as a reduction in emulsion loading efficiency. In general, the emulsion degraded by 30–60% after 7 days under light conditions. The emulsions stabilized by SPI and LEC had good degradation resistance, and their degradation ratios were below 40%, which were lower than that of the pure oil phase. In contrast, the degradation ratios of the stable emulsions of small molecular emulsifiers T80, S80, and SDS were higher than that of the pure oil phase. According to the error analysis of repeated experiments, the deviation was not obvious, indicating that the curcumin-load ratio had good repeatability with the storage time.

Emulsifiers with larger molecular weights can effectively protect the curcumin by forming a thicker emulsifier shell owing to their larger steric hindrance, thereby reducing the degree of degradation [1]. Furthermore, LEC can inhibit the chemical degradation of the active substance by trapping the bioactive substance inside the emulsifier layer and the oil phase, which prevent contact with the pro-oxidant in the water phase [33]. LEC had been widely used to construct three-dimensional supramolecular networks. It mainly self-assembled with active substances or lipids through non-covalent interactions, such as electrostatic interactions, hydrogen bonds, van der Waals forces, hydrophobic interactions, etc. [16]. Through homogenization and stirring, SPI can form a complex with curcumin through hydrophobic interaction [34], and this potential interaction can improve the loading effect of SPI on curcumin, thereby preventing degradation. This is one of the reasons why its degradation ratio was lower than that of the pure oil phase. Proteins can also self-assemble with carbohydrates or other proteins through chemical interactions, such as hydrogen bonds, hydrophilic groups, and ionic bonds [27].

In addition, the average droplet diameter under light conditions was not affected, and no demulsification occurred. The decrease in the load of the photosensitizer was not caused by the destruction of the stability of the emulsion. The emulsion should be stored under dark conditions in practical applications.

### 3.2. Freeze-Thaw Stability

The dispersed lipid droplets and their behavior play a key role in determining the stability of the emulsion during the freeze-thaw process [21]. Pure lipids need to nucleate by using dust or flaws on the inner wall of the container. Lipid droplets are usually nucleated by the principle of homogeneous nucleation due to their small particle size. Thus, the crystallization temperature of the droplets after emulsification is lower than that of pure lipid. The crystallization temperature of the pure lipid MCT used in this experiment was approximately −34 °C, and the lowest freeze-thaw treatment was performed at −20 °C. Therefore, the emulsion in this experiment only crystallized in the aqueous phase.

The demulsification phenomenon after freezing and thawing of the emulsion originates from the destruction of the droplets when the ice crystals are formed and the subsequent reformation of the droplets after the ice crystals melt. When the emulsion is frozen, the amount of liquid water decreases with the increasing volume fraction of the dispersed phase; the droplets are strained from the expanding ice phase [35]. The sources of such mechanical stress and strain are usually the deformation caused by the Brownian motion between the droplets and the collision caused by the growth of ice crystals. The difference in the resistance of the droplets to deformation causes the lipids to partially or completely coalesce after freezing and thawing, leading to a decrease in the number of droplets and an increase in the average size.

**Figure 4:**
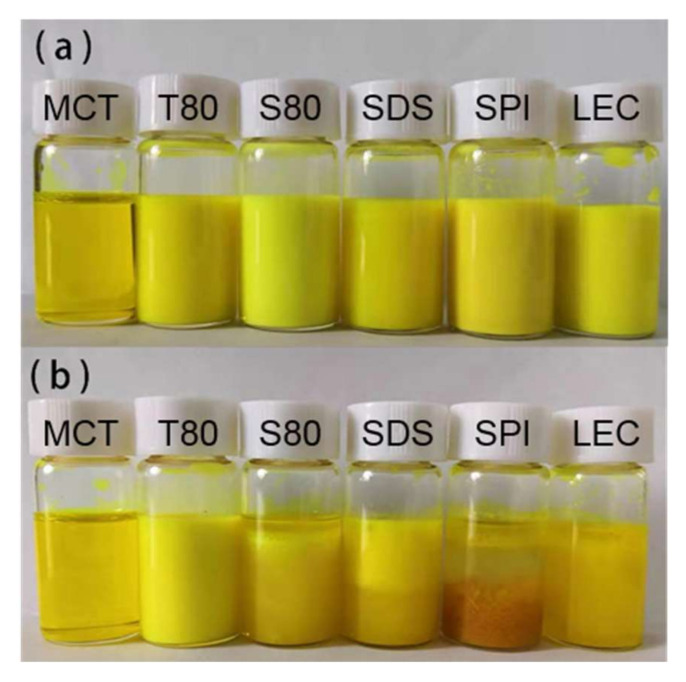
Digital photos of the emulsions before (**a**) and after (**b**) the freeze-thaw treatment.

**Figure 5:**
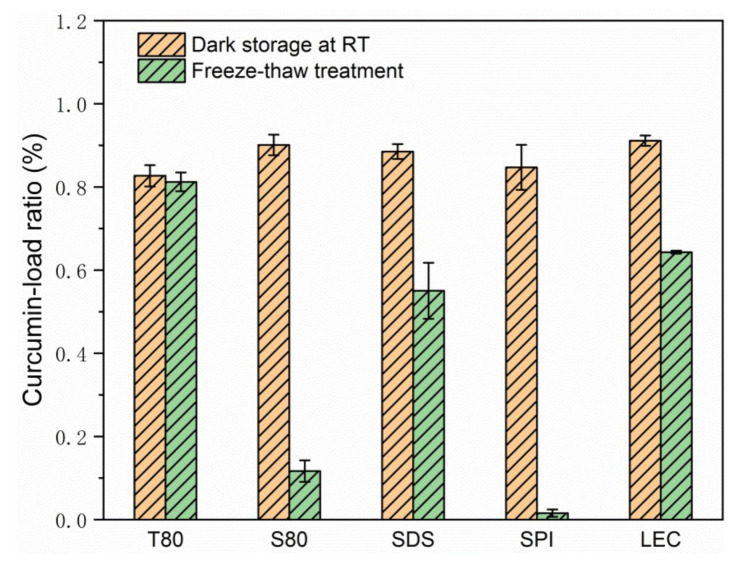
Curcumin-load ratio of emulsions after 3 days of storage at room temperature or during the freeze-thaw treatment.

According to Figure 4 and Figure 5, Figure S3, and Table 2, after the freeze-thaw cycle treatment, the emulsions of each group showed obvious demulsification, except for the emulsion stabilized by T80.

The S80 stable emulsion appeared as oil precipitated in the upper layer, and the lower layer was slightly transparent water. Considering its curcumin-load ratio of less than 10% and the sharply increased particle size, the formation of water phase ice crystals during the freezing process caused droplet cracking, and a large amount of oil carried curcumin to precipitate. During the melting of ice crystals, due to the lipophilic nature of emulsifier S80, which is typically used as a water in oil (W/O) emulsifier, the re-emulsifying ability was poor when there is no high energy input, and the macroscopic performance showed the demulsification of oil phase aggregation phenomenon.

The upper layer of the SDS stable emulsion was emulsion-like, and the lower layer was slightly transparent water. According to the curcumin-load ratio, of about 60% of its emulsion-like part and the size of the droplets, it was speculated that the droplets ruptured during freezing. Parts of curcumin were loaded with the emulsifier to form micelles and diffused into the water phase because of the small molecular weight of SDS and good mobility. Given that the acidity coefficient (pKa) value of curcumin in water was 8.1 [8], its main existence forms are cur^0^ and enolate ion cur^−1^ under neutral conditions. According to electrostatic interaction, the curcumin population combined with anionic SDS micelles was neutral enol cur^0^ [36], and some of the cur^−1^ micelles diffused in the water to undergo hydrolysis, resulting in the decrease in coverage during the freeze-thaw cycle. After the ice crystals melted, the strong emulsifying ability provided by the SDS was easy to move and could form an emulsion again, but the particle size was larger. According to the density, the emulsion part was on the upper side of the water phase. However, due to the lack of applied mechanical force and the reduction of SDS content, the emulsion had a smaller volume. Macroscopically, the water phase carrying curcumin remained in the lower part of the sample.

The uppermost layer of the SPI stable emulsion was the oil phase, the middle layer was a yellowish transparent emulsion, and the lower layer was a flocculent solid precipitate. As the droplets ruptured during freezing, the oil phase separated, and the interaction between the proteins increased the viscosity of the sample. Given that globular proteins tended to form a 2D cross-linked molecular network at the oil–water interface [23], a microgel structure was formed at the interface. The curcumin in the oil phase was partially hindered by the protein during movement and had hydrophobic interaction with it [34,37], thereby remaining inside the gel structure of the protein molecule. The water in the gel was solid during the freeze-thaw process, which made the proteins more tightly connected. Thus, the protein eventually precipitated as a flocculent solid in the lower part of the sample. This phenomenon was consistent with those of previous studies on protein emulsions, which appeared to thicken after oiling and cooling at room temperature after the freeze-thaw treatment [21]. Considering the droplet diameter of the SPI stable emulsion, SPI formed a stable emulsifier shell before the freeze-thaw treatment, but the strength was low, and the droplets could not be protected from ice crystal damage under freezing conditions. The high milking value and the large amount of oiling caused by the aging problem of the SPI prepared emulsion sample affected its freeze-thaw stability. Moreover, the volume of the unfrozen water phase was increased by adding anti-freeze compounds, such as glucose, thereby allowing the oil droplets to better reorganize to resist the stress exerted by the swollen ice and to improve the freeze-thaw stability of the emulsion [38]. In addition, a thicker interfacial film could also be produced through the layer-by-layer deposition method to improve the damage resistance. Noshad et al. [20] prepared SPI–octenyl succinate starch (OSA) starch–chitosan multilayer emulsion that effectively improved the freeze-thaw stability compared with the SPI single-layer emulsion. Silva et al. [39] used layer-by-layer electrostatic technology to deposit chitosan and alginate polyelectrolyte layer by layer to improve the encapsulation efficiency of curcumin.

A small part of the oil phase precipitated in the upper layer of the LEC stable emulsion, and the lower part was a slightly turbid emulsion. The emulsion layering phenomenon was similar to that of the SDS stable emulsion, but no obvious water-like layering was observed, which could be attributed to the similar molecular weights of LEC and SDS. LEC had a smaller molecular weight than T80, S80, and SPI. Thus, LEC could still coat certain oil phase droplets in the water phase during the freeze-thaw process and maintain part of the emulsion state. However, compared with SDS, the LEC hydrophilic head group and the hydrophobic tail group were larger and longer, leading to a slower movement, thereby increasing the droplet size after melting. Nonetheless, the curcumin-load ratio was higher. LEC and T80 had similar molecular structures, but they showed different performances in terms of freeze-thaw stability of emulsions, which was related to LEC’s higher fat solubility. A large amount of LEC was dissolved in the lipid phase, which was broken by the ice crystals. As the lipid phase floated, the LEC content in the emulsion decreased, thereby reducing the sample’s re-emulsifying ability, and finally resulting in the emulsion’s demulsified state. This result was similar to that obtained for the fat-soluble S80 emulsifier, which was consistent with the findings of the greatly increased particle size of the two emulsions in Table 2. Donsì et al. [22] suggested that the instability of LEC-stabilized emulsions after freeze-thaw treatment could be attributed to the formation of ice crystals during the freezing of the emulsion and the frequent occurrence of droplet aggregation. According to the coalescence transition state theory [40], the migration of lecithin molecules on the droplet surface of low melting point lipids might affect the degree of coalescence. The structure and thermal stability of the LEC system mainly depended on the type and strength of the chemical interaction between the lipid molecules and the LEC. Accordingly, LEC had static and dynamic self-assembly mechanisms [16].

### 3.3. Thermal Stability

For the characterization of the thermal stability of the emulsion, the heating of the emulsion sample is in an oven, so the temperature of 120 °C is the ambient temperature. In the observation of the experiment, no boiling of the emulsion sample was found. Therefore, on the one hand, it is believed that the addition of the oil phase may increase the boiling point of the emulsion. On the other hand, it is believed that because the ambient temperature is 120 °C, the emulsion sample itself may not reach 120 °C. For the evaporation of emulsion at high temperature, the emulsion sample bottle is fastened. Therefore, during the heating process, the water phase will evaporate to a certain extent, but due to the sealing of the sample bottle, the water vapor cannot escape, and finally remains in the sample bottle in the form of liquid water.

Heat treatments were conducted at 60 °C, 90 °C, and 120 °C for each group of emulsions. Table 3 and Figure S4 shows that except for SPI, the droplet size of each type of emulsion was less affected by heating conditions, and no obvious change was observed in particle size and demulsification. After the heat treatment of emulsions at different temperatures and durations, curcumin showed different degrees of degradation.

**Figure 6:**
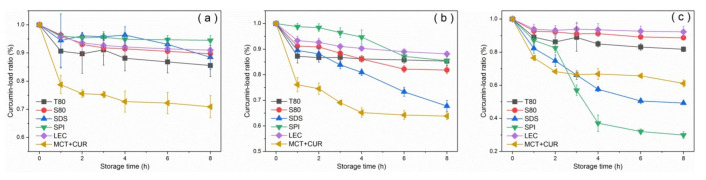
Curcumin-load ratio of emulsions after heat treatment at 60 °C (**a**), 90 °C (**b**), and 120 °C (**c**).

Figure 6 shows the curcumin-load ratio of each emulsion group after heat treatment. The degradation degree of the pure oil phase at 60 °C was about 20% at the maximum, whereas the degradation ratio of the emulsion was low, and the curcumin-load ratio of 90% was maintained. The pure oil phase was degraded by more than 30% at 90 °C for 8 h, and the degree of degradation was the largest in the first hour. Similar results were obtained by Wang et al. [41], who reported that curcumin dissolved in MCT decomposed by 10% after dark heat treatment at 90 °C for 1 h. According to Gibbs and Langmuir adsorption isotherms, the emulsifying ability of an emulsifier is usually affected by its adsorption rate and its ability to reduce interfacial tension. [42]. The adsorption rate was determined by its molecular characteristics, including the molecular weight, number, and position of hydrophilic and hydrophobic groups [17]. Generally, the smaller the volume and mass are, the greater the adsorption rate is. Comparing the curcumin-load properties at 90 °C, SPI had a better ability to reduce interfacial tension than SDS and had a larger adsorption rate. Sanidad et al. [2] believed that the emulsion system improves the stability of curcumin, because contact with hydrophilic substances (such as OH^−^), which causes degradation in the aqueous phase, can be avoided by dispersing the curcumin in the hydrophobic core.

Within the first 2 h at a heating temperature of 120 °C, SPI aggregation formed a thicker interface layer to maintain the stability of the emulsion. However, after 2 h, the formation and adsorption of SPI aggregates [19] formed a bridge between the droplets, which greatly increased the droplet size and caused the leakage and degradation of curcumin from the lipid. This inference was consistent with the increase in particle size of the emulsion. Natural glycinin can self-assemble in an aqueous solution. The size of the assembly increased with the increase of protein concentration and the decrease of pH value, and the process was reversible [27]. Chen et al. [43] found that under low concentration or short heating time, glycinin would form irregular spherical particles. Moreover, as the protein concentration and heating time increased, these particles would randomly aggregate and might form a gel. At 120 °C, the SPI stabilized emulsion had obvious demulsification after 3 h, which can be attributed to the thermal instability of the protein and the degradation of the curcumin. Under heating, the two main proteins present in SPI (β-conglycinin and glycinin) were denatured. The different emulsification and coating properties displayed by SPI at 90 °C and 120 °C were related to the different denaturation temperatures of the two proteins [19]. Lakemond et al. [26] indicated that heating at above 95 °C could cause a large amount of unfolding of the two globulins, thereby exposing the sulfhydryl and hydrophobic groups, which induced the interaction between the subunits and the polypeptide chain and led to the formation of soluble complexes between the subunits. According to the study of sodium caseinate-curcumin interaction [37] and the discussion on the formation of the SPI–CUR complex [34], it can be considered that heating will cause the hydrophobic groups of SPI to be exposed. Therefore, it can bind to curcumin molecules through hydrophobic interactions. The curcumin molecules were separated from the MCT oil phase and dissolved in the water phase with the protein, thereby promoting the thermal degradation and hydrolysis of curcumin.

Under the heating at 90 °C and 120 °C, the curcumin of SDS stable emulsion showed obvious degradation and increased with heating time. After curcumin molecules were introduced into the emulsion, they were generally considered to be located inside the hydrophobic interior of the oil droplets due to the lipophilic nature of curcumin. According to Anuchapreeda et al. [31], curcumin had polar groups, which can be located near the oil–water interface in emulsions with LEC as the emulsifier [2]. Kumar et al. [44] found that in curcumin micelles, the conjugated hydrocarbon portion of curcumin could interact with the hydrophobic portion of surfactant (DDAB). Thus, it was located in the micelle of the surfactant head polar section. Therefore, it is believed that curcumin is mainly located in the interface layer of the emulsion due to its polarity. In micelles, the polar groups are in the palisade layer (nearby the Stern layer) with the hydrophobic portions in the hydrophobic interior. Because curcumin is located at the oil–water interface, the molecular weight of the emulsifier will significantly affect the degradation of curcumin at the interface. Therefore, the curcumin blended in the SDS stable emulsion has obvious thermal decomposition. This speculation coincided with the decrease in absorbance of SDS stabilized emulsion after the freeze-thaw treatment.

Emulsions stabilized by T80, S80, and LEC showed high thermal stability and protected curcumin. According to the error analysis of repeated experiments in Figure 6, it could be considered that the stable emulsions of SPI and LEC were greatly affected by the environment and had poor repeatability.

### 3.4. Antibacterial Effect

**Figure 7:**
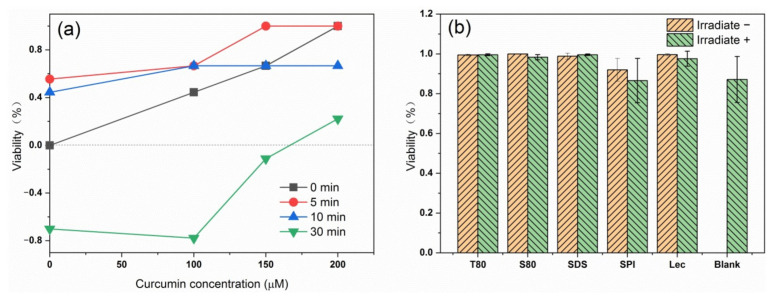
The antimicrobial effect of the emulsions prepared with T80 on *E. coli* under different light times and curcumin concentrations (**a**); antibacterial effect of emulsions containing different emulsifiers against *E. coli* (**b**).

First, T80 was used as an emulsifier to compare the antibacterial effect of emulsion against *E. coli* under different light times and photosensitizer concentrations. Figure 7a shows that the increase of curcumin content and the decrease of light time increased the antibacterial effect of the photodynamic treatment against *E. coli*. Therefore, the curcumin concentration of 150 µM and the light time of 5 min were selected as experimental conditions for the next experiment. According to the monitoring of the antibacterial effect of emulsions containing different emulsifiers in Figure 7b, adding a photosensitizer or selecting blue light irradiation had a significant antibacterial effect on the samples compared with the blank non-illuminated bacterial samples. However, when blue light irradiation was used in combination with curcumin, the bactericidal effect on bacteria was partially increased compared with simple light irradiation, but the increase was not obvious. Emulsions containing protein SPI had poor bactericidal properties. The properties of proteins affected the antibacterial effect against *E. coli*. In the sterilization test, all types of nanoemulsions loaded with curcumin could achieve sterilization under blue light, and the sterilization ratio increased to more than 85% by adjusting the emulsion concentration and light time. Except for SPI, the type of emulsifier had no significant effect on the bactericidal effect of the photosensitive emulsion.

## 4. Conclusions

MCT was used as the oil phase, and curcumin was used as the photosensitizer. The stability, curcumin-load ratio, and bactericidal effect of emulsions prepared by different emulsifiers were studied. Emulsions stabilized by T80, S80, SDS, and LEC had better stability during storage. The surface charge of SDS was large, and the repulsive force between droplets increased. Emulsions stabilized by SPI and LEC could effectively improve curcumin’s anti-degradability under light conditions. Emulsions stabilized by T80 showed the highest emulsion stability during freeze-thaw treatment. Emulsions stabilized by T80, S80, and LEC had better stability during heat treatment. In summary, T80 and LEC resulted in good stability, curcumin-load effect, and bactericidal properties of the emulsion under heating or at room temperature. T80 and LEC have the potential to be used in large quantities as emulsifiers for curcumin-loaded bactericidal emulsions. The differences between emulsifiers were due to emulsifier’s molecular structures and molecular weights. A large molecular weight and the proper size ratio of the head and tail groups can improve the stability of the emulsion and the protective effect on curcumin to a certain extent. Through the discussion of emulsion stability and curcumin-load ratio under different conditions, such as room temperature, light, heating, and freezing, new considerations for the future processing conditions of such emulsions in practical applications are provided. A food-grade emulsion loaded with antibacterial photosensitizer is a new idea that can be used for the production of many types of food commodities.

## Figures and Tables

**Table 1:** The parameters of various emulsions at different storage times (25 °C).

Emulsifier Type	Storage Time (Day)	Mean Droplet Diameter (Nm)	Polydispersity Index	ζ-Potential (mV)
T80	0	155 ± 0.7 ^j^	0.10 ± 0.01 ^d^	−30.9 ± 0.6 ^e^
	3	159 ± 1.4 ^i^	0.11 ± 0.01 ^d^	−22.9 ± 0.9 ^i^
	7	188 ± 3.2 ^f^	0.22 ± 0.01 ^c^	−21.0 ± 1.0 ^i^
S80	0	322 ± 2.4 ^b^	0.29 ± 0.02 ^b^	−30.1 ± 0.5 ^e^
	3	293 ± 4.3 ^d^	0.23 ± 0.01 ^c^	−29.1 ± 0.5 ^f^
	7	299 ± 4.1 ^c^	0.22 ± 0.00 ^c^	−27.3 ± 1.2 ^g^
SDS	0	108 ± 0.9 ^k^	0.11 ± 0.01 ^d^	−53.8 ± 2.1 ^a^
	3	109 ± 1.8 ^k^	0.12 ± 0.01 ^d^	−46.2 ± 1.4 ^b^
	7	110 ± 0.7 ^k^	0.10 ± 0.01 ^d^	−44.9 ± 1.2 ^c^
SPI	0	199 ± 2.7 ^e^	0.23 ± 0.01 ^c^	−33.9 ± 0.7 ^d^
	3	-	-	−22.5 ± 0.7 ^i^
	7	4400 ± 869 ^a^	0.62 ± 0.05 ^a^	−12.4 ± 0.7 ^j^
LEC	0	160 ± 1.1 ^h^	0.07 ± 0.01 ^e^	−35.0 ± 1.4 ^d^
	3	184 ± 1.6 ^g^	0.21 ± 0.01 ^c^	−25.9 ± 0.1 ^h^
	7	164 ± 2.5 ^h^	0.08 ± 0.01 ^d^	−27.8 ± 0.2 ^g^

Different superscripts (a–k) represent significant difference at *p* < 0.05 level among the same column (*n* = 3).

**Table 2:** The parameters of various emulsions after 3 days of freeze-thaw treatment.

Emulsifier Type	Mean Droplet Diameter (Nm)	Polydispersity Index	ζ-Potential (mV)
T80	174 ± 2.7 ^e^	0.09 ± 0.01 ^c^	−27.4 ± 1.2 ^c^
S80	506 ± 6.8 ^c^	0.40 ± 0.02 ^b^	−36.6 ± 0.9 ^b^
SDS	743 ± 75.7 ^b^	0.63 ± 0.08 ^a^	−77.8 ± 1.7 ^a^
SPI	246 ± 54.0 ^d^	0.39 ± 0.04 ^b^	−22.7 ± 0.5 ^d^
LEC	1639 ± 165.3 ^a^	0.36 ± 0.05 ^b^	−32.8 ± 2.2 ^b^

Different superscripts (a–e) represent significant difference at *p* < 0.05 level among the same column (*n* = 3).

**Table 3:** The parameters of various emulsions after 2 h of heat treatment.

Emulsifier Type	Heat Treatment Temperature (°C)	Mean Droplet Diameter (nm)	Polydispersity Index
T80	60	155 ± 1.8 ^i^	0.09 ± 0.01 ^d^
	90	156 ± 1.1 ^i^	0.09 ± 0.01 ^d^
	120	169 ± 2.3 ^g^	0.04 ± 0.00 ^f^
S80	60	288 ± 2.1 ^d^	0.23 ± 0.02 ^b^
	90	292 ± 2.5 ^c^	0.23 ± 0.01 ^b^
	120	305 ± 1.9 ^b^	0.23 ± 0.01 ^b^
SDS	60	109 ± 0.3 ^l^	0.13 ± 0.01 ^d^
	90	111 ± 0.5 ^k^	0.10 ± 0.02 ^d^
	120	125 ± 2.3 ^j^	0.09 ± 0.01 ^d^
SPI	60	175 ± 1.0 ^f^	0.08 ± 0.01 ^e^
	90	256 ± 1.4 ^e^	0.23 ± 0.01 ^b^
	120	6003 ± 41.6 ^a^	0.58 ± 0.08 ^a^
LEC	60	163 ± 2.4 ^h^	0.08 ± 0.03 ^e^
	90	162 ± 0.4 ^h^	0.09 ± 0.01 ^d^
	120	177 ± 0.3 ^f^	0.17 ± 0.02 ^c^

Different superscripts (a–l) represent significant difference at *p* < 0.05 level among the same column (*n* = 3).

## Data Availability

The data used to support the findings of this study are available from the corresponding author upon request.

## Supplementary Materials

The following are available online at https://www.mdpi.com/2304-8158/10/1/149/s1, Figure S1: Standard curve of curcumin absorbance, Figure S2: Droplet size distributions for emulsions as a function of storage time: (a) fresh emulsion, (b) dark storage at 25 °C for 3 days, (c) dark storage at 25 °C for 7 days, Figure S3: Droplet size distribution of emulsions after freeze-thaw treatment, Figure S4: Droplet size distributions for emulsions as a function of heating temperature: (a) 60 °C, (b) 90 °C, (c) 120 °C.
