# Supplementary material for: Effects of Emulsifier Type and Post-Treatment on Stability, Curcumin Protection, and Sterilization Ability of Nanoemulsions
Source: Foods. 2021 Jan 13;10(1):149. doi: 10.3390/foods10010149 (PMC7828267; doi:10.3390/foods10010149)
Supplement: Supplementary file 1 [file foods-10-00149-s001.pdf]

Supplementary Materials

Effects of emulsifier type and post-treatment on stability, curcumin protection, and sterilization ability of nanoemulsions

Rui Li [1], Qiangsheng Fang [1], Peihong Li [1], Chunling Zhang [1,*], Yuan Yuan [2], and Hong Zhuang [2,]

1 School of Materials Science and Engineering, Jilin University, Changchun, 130022, P.R. China; ruili18@mails.jlu.edu.cn (R.L.); fangqs19@mails.jlu.edu.cn (Q.F.); liph18@mails.jlu.edu.cn (P.L.);

2 College of Food Science and Engineering, Jilin University, Changchun, 130062, P.R. China; yuan_yuan@jlu.edu.cn (Y.Y.)

* Correspondence: clzhang@jlu.edu.cn (C.Z.); Tel.: +86-431-8509-5170(C.Z.)

Corresponding Author

*E-mail: clzhang@jlu.edu.cn

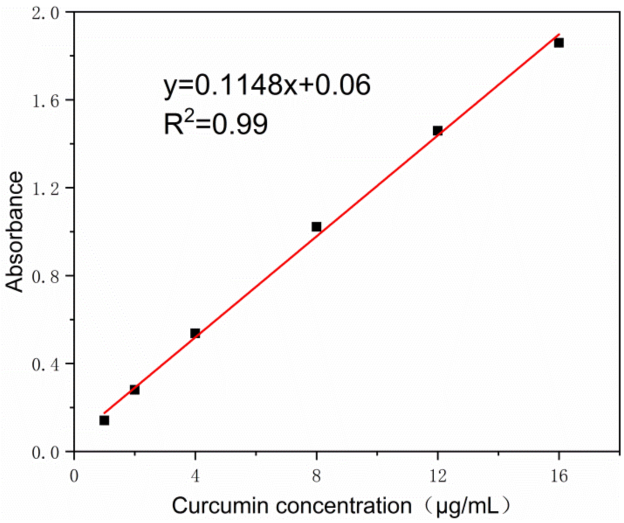

Figure S1. Standard curve of curcumin absorbance.

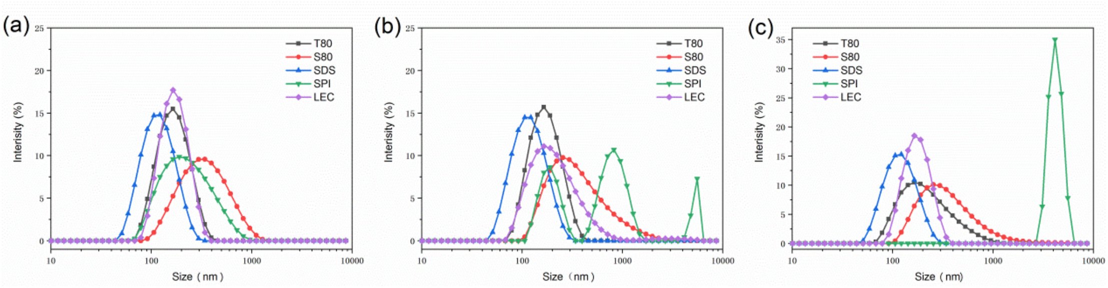

Figure S2. Droplet size distributions for emulsions as a function of storage time：(a) fresh emulsion, (b) dark storage at 25 °C for 3 days, (c) dark storage at 25 °C for 7 days.

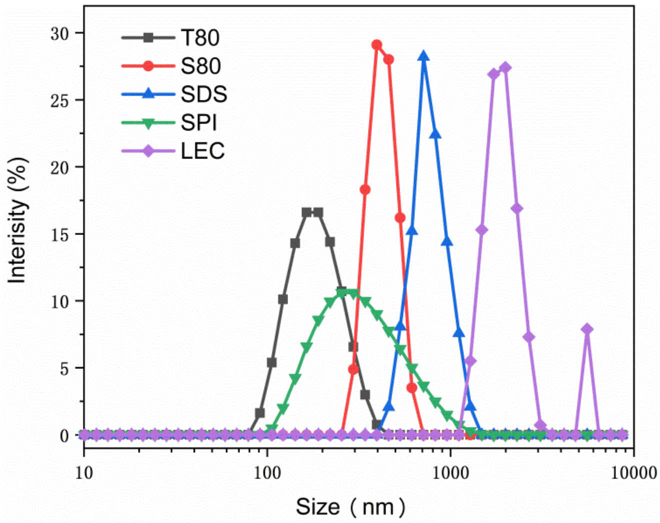

Figure S3. Droplet size distribution of emulsions after freeze-thaw treatment.

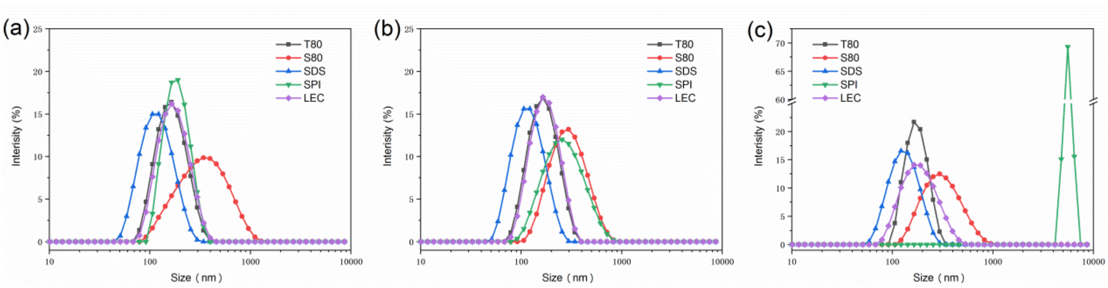

Figure S4. Droplet size distributions for emulsions as a function of heating temperature: (a) 60 °C, (b) 90 °C, (c) 120 °C.
